# Solid‐State Ligand‐Exchange Fabrication of CH_3_NH_3_PbI_3_ Capped PbS Quantum Dot Solar Cells

**DOI:** 10.1002/advs.201500432

**Published:** 2016-02-18

**Authors:** Jiajun Peng, Yani Chen, Xianfeng Zhang, Angang Dong, Ziqi Liang

**Affiliations:** ^1^ Department of Materials ScienceFudan UniversityShanghai200433P.R. China; ^2^ Department of ChemistryFudan UniversityShanghai200433P.R. China

**Keywords:** CH_3_NH_3_PbI_3_ perovskites, layer‐by‐layer method, ligand exchange, PbS quantum dots, photovoltaics

## Abstract

**CH_3_NH_3_PbI_3_ capped PbS** colloidal quantum dots have been successfully fabricated by solid‐state ligand exchange from oleate and oleylamine capped PbS. The optimal solar cells made by layer‐by‐layer solution deposition give a high power conversion efficiency of 4.25% with an impressive short‐circuit photocurrent density of 24.83 mA cm^−2^.

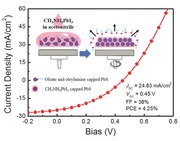

Lead sulfide (PbS) colloidal quantum dots (CQDs), which possess tunable near‐infrared absorption,^1^ multiple exciton generation effect,^2^ and solution processability,^3^ have emerged as attractive candidates for thin film photovoltaic applications.^4^ The surface ligands of CQDs play a crucial role in determining the optoelectronic properties of CQD solar cells.^5^ Generally, CQDs that bear long‐chain organic ligands offer the solubility, yet those ligands unavoidably form insulating barriers of charge transport between CQDs. Therefore, short‐chain ligands are employed to shrink the inter‐spacing of crystals and reduce surface trap states, both of which favor charge transport.^6^ Currently, 1,2‐ethanedithiol (EDT) and 3‐mercaptopropionic acid (MPA) are two of most widely used chalcogenide‐based ligands for PbS solar cells,^7^ which yielded power conversion efficiencies (PCEs) over 3%^8^ and 8%,^9^ respectively.

Yet the PCEs of PbS‐based solar cells seem difficult to further improve because of their several intrinsic obstacles. One is that traditional ligands such as EDT and MPA do not contribute to optical absorbance, thereby limiting short‐circuit photocurrent density (*J*_sc_). The other is that the thickness of CQD photovoltaic layers is limited by short carrier diffusion lengths between CQDs, which causes serious charge recombination.^10^ Thus, novel ligands, for instance, which possess a long carrier diffusion length and a complementary optical spectrum with PbS, are urgently needed to improve CQD solar cell performance.

Recent years have witnessed unprecedented development of organohalide perovskites (CH_3_NH_3_PbX_3_, X = I, Br, Cl) in thin film solar cells,^11^, ^12^, ^13^, ^14^, ^15^, ^16^ owing to their magnificent properties such as long hole and electron diffusion lengths,^17^, ^18^ broad visible absorption range,^19^ and high charge mobility.^20^, ^21^ More importantly, CH_3_NH_3_PbI_3_ shows perfect lattice matching with PbS CQDs.^22^ In this regard, CH_3_NH_3_PbX_3_ can be considered as a promising ligand for PbS. Very recently, CH_3_NH_3_PbI_3_ perovskites were successfully used as stable capping ligands for a range of CQDs—PbS, CdS, InP, and CdSe—via solution ligand‐exchange reaction, resulting in efficient electronic passivation for highly luminescent CQDs.^23^ More recently, PbS‐in‐perovskite solids were fabricated through in situ epitaxial growth process in solution phase to attain excellent carrier transport between perovskites and PbS.^22^ Most recently, such PbS‐in‐perovskite solids were combined with EDT capped PbS (namely, PbS−EDT) CQDs and then applied in hybrid nanostructured solar cells, obtaining a high PCE of 8.95%.^24^ However, all these PbS—CH_3_NH_3_PbI_3_ CQDs were synthesized through solution‐phase ligand exchange and their solar cells involved the fabrication of multiple‐layered device structures.

Herein, we report a solid‐state ligand exchange method to obtain PbS CQDs with CH_3_NH_3_PbI_3_ capping ligands (namely, PbS—CH_3_NH_3_PbI_3_), followed by a layer‐by‐layer (LbL) solution deposition method to fabricate relatively thick film with controllable thickness as the active layer in solar cells. A highly volatile solvent, acetonitrile, is used for CH_3_NH_3_PbI_3_ in successive steps of ligand exchange, spin‐coating, and solvent rinsing. Such solvent strategy of using acetonitrile is crucial to obtain smooth, continuous, and thick films, accompanied by complete replacement of original ligands and removal of extra CH_3_NH_3_PbI_3_. The resulting PbS—CH_3_NH_3_PbI_3_ exhibits a broadened absorption spectrum in both visible and near‐infrared wavelengths, and efficient charge transfer between quantum dots and perovskites. Moreover, cascade energy alignment can be achieved among PbS, CH_3_NH_3_PbI_3_, and electron‐selective layer (TiO_2_) to facilitate charge transport. The optimal solar cell reaches a PCE of 4.25% under AM 1.5 G light irradiation with an impressive *J*_SC_ of 24.83 mA cm^−2^. This PCE is remarkably higher than those reported values of traditional EDT‐capped PbS solar cells (≈3%).^7^, ^8^, ^25^, ^26^

PbS CQDs were first synthesized by using lead(II) oleate with bis(trimethylsilyl) sulfide, and the original capping ligands are oleate and oleylamine.^1^ Prior to solid‐state ligand exchange, the CQDs were dispersed in octane solvent. As‐synthesized PbS CQDs were structurally characterized by transmission electron microscope (TEM) and ultraviolet‐visible‐near‐infrared (UV–vis–NIR) spectrophotometer. As displayed in TEM image (**Figure** **1**a), uniform‐sized PbS are obtained with an average diameter of 2.5 nm and a lattice fringe of 3.5 Å. Moreover, energy dispersive X‐ray spectroscopy (EDX) spectrum (Figure S1 in the Supporting Information) shows that the atomic percentage of lead to sulfur in PbS is nearly 1:1. Figure 1b displays the optical absorption spectrum of PbS in octane solution, which exhibits a near‐infrared peak at 824 nm, giving an optical bandgap of ≈1.50 eV. Cyclic voltammetry (CV) was further applied to determine the energy levels of PbS. Figure S2 (Supporting Information) shows that the valence and conduction bands of PbS are −5.19 and −3.65 eV, respectively, yielding an electrochemical bandgap of 1.54 eV, which is very close to the optical bandgap.

**Figure 1:**
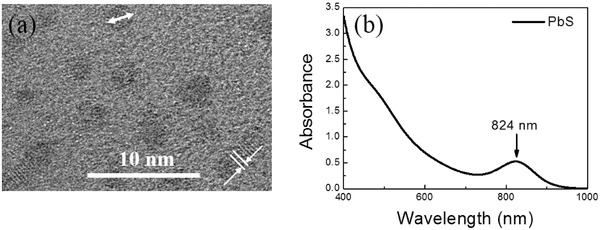
Structural characterization of PbS CQDs capped with a mixture of oleate and oleylamine. a) TEM image showing an average size of 2.5 nm in diameter. b) Optical absorption spectrum in octane solution.

Next, perovskite CH_3_NH_3_PbI_3_ was introduced as a replacing ligand of PbS CQDs via solid‐state ligand exchange. **Scheme** **1** outlines the fabrication process of the PbS—CH_3_NH_3_PbI_3_. First, the PbS with mixed ligands of oleate and oleylamine was spin‐coated from the octane solution (20 mg mL^−1^) onto glass substrate. Second, a saturated CH_3_NH_3_PbI_3_ solution in acetonitrile was drop‐casted onto the entire PbS layer and remained for 1 min to achieve complete ligand exchange (Scheme 1a). Spin‐coating was then applied to remove the excess solution (Scheme 1b). Lastly, two consecutive rinsing steps were applied with acetonitrile and octane to remove extra perovskites and organic ligands, each followed by spin‐coating. These three steps were repeated several times through LbL methods to obtain the proper thickness of brown and smooth films. Note that we have initially attempted to use common solvents such as *N*,*N*‐dimethylformamide (DMF) and *N*‐methylformamide (MFA) for preparing CH_3_NH_3_PbI_3_ solution; yet owing to their low volatility and high solubility for CH_3_NH_3_PbI_3_, both solvents tend to redissolve the majority of PbS—CH_3_NH_3_PbI_3_ in the fabrication process, yielding cracked and discontinuous films.

**Scheme 1:**
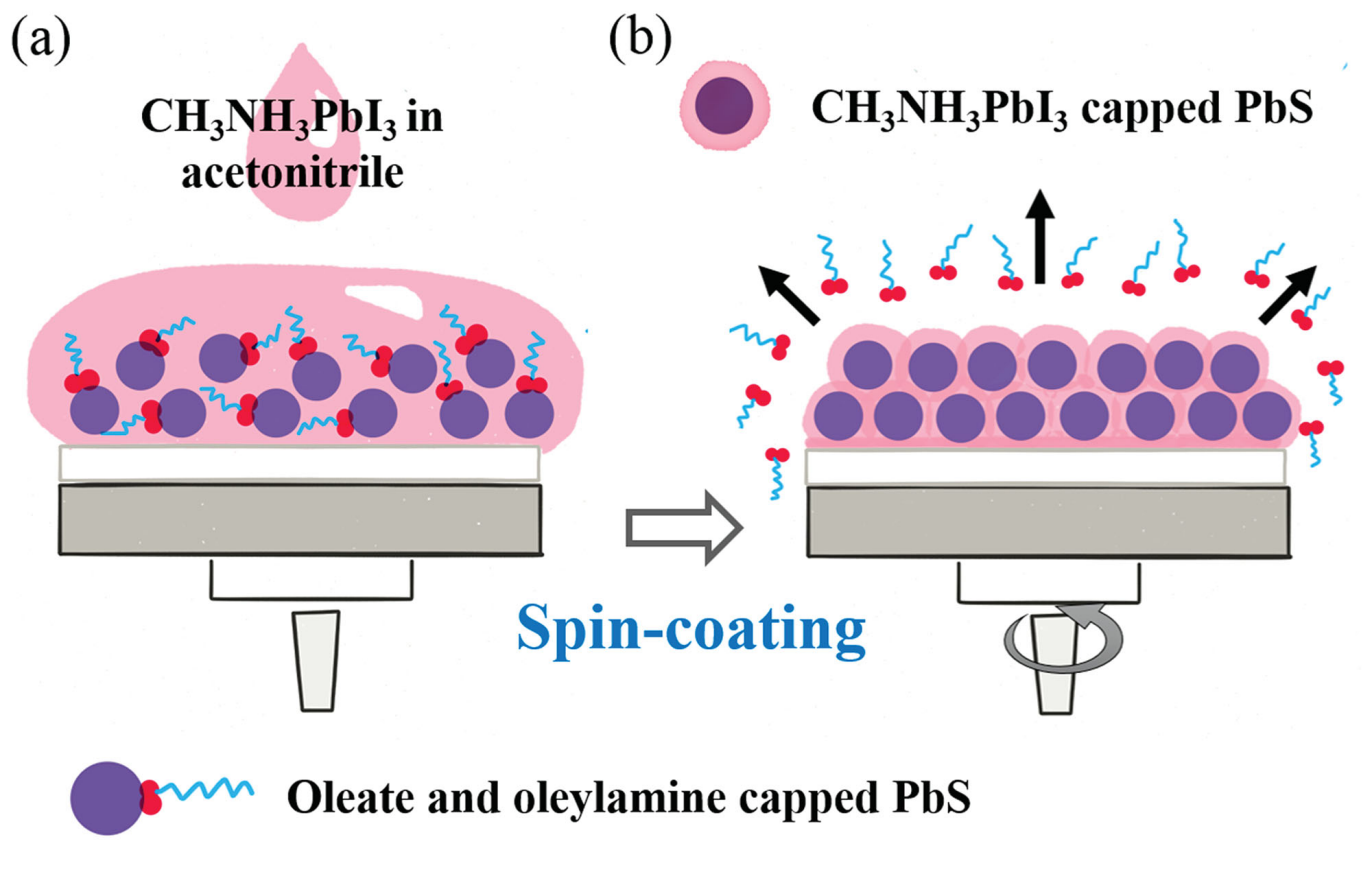
Schematic fabrication of PbS—CH_3_NH_3_PbI_3_ CQDs via ligand‐exchange during spin‐coating deposition.

Such ligand‐exchange process of making PbS—CH_3_NH_3_PbI_3_ CQDs was fully characterized, as shown in **Figure** **2**. Fourier‐transform infrared (FTIR) spectroscopy was first used to confirm the success of ligand exchange process. As shown in Figure 2a, oleate and oleylamine capped PbS CQDs display strong symmetrical and asymmetrical CO_2_^−^ vibration bands at 1405 and 1548 cm^−1^, and C−H stretching peaks at 2854 and 2926 cm^−1^, respectively, both of which are characteristic of the carboxylate functional group in oleic acid.^23^ By contrast, these CO_2_^−^ and C—H peaks are absent in the PbS—CH_3_NH_3_PbI_3_ when the loading time of PbS film in CH_3_NH_3_PbI_3_ solution is 1 min. This indicates that perovskite has successfully replaced the original mixed ligands. TEM image was further characterized to ensure the formation of PbS—CH_3_NH_3_PbI_3_ CQDs as shown in Figure S3 in the Supporting Information. After ligand exchange, the size and lattice fringe of PbS—CH_3_NH_3_PbI_3_ CQDs remain nearly the same. Meanwhile, with the aid of CH_3_NH_3_PbI_3_ ligands, PbS CQDs tend to inter‐connect and form a continuous solid matrix.

**Figure 2:**
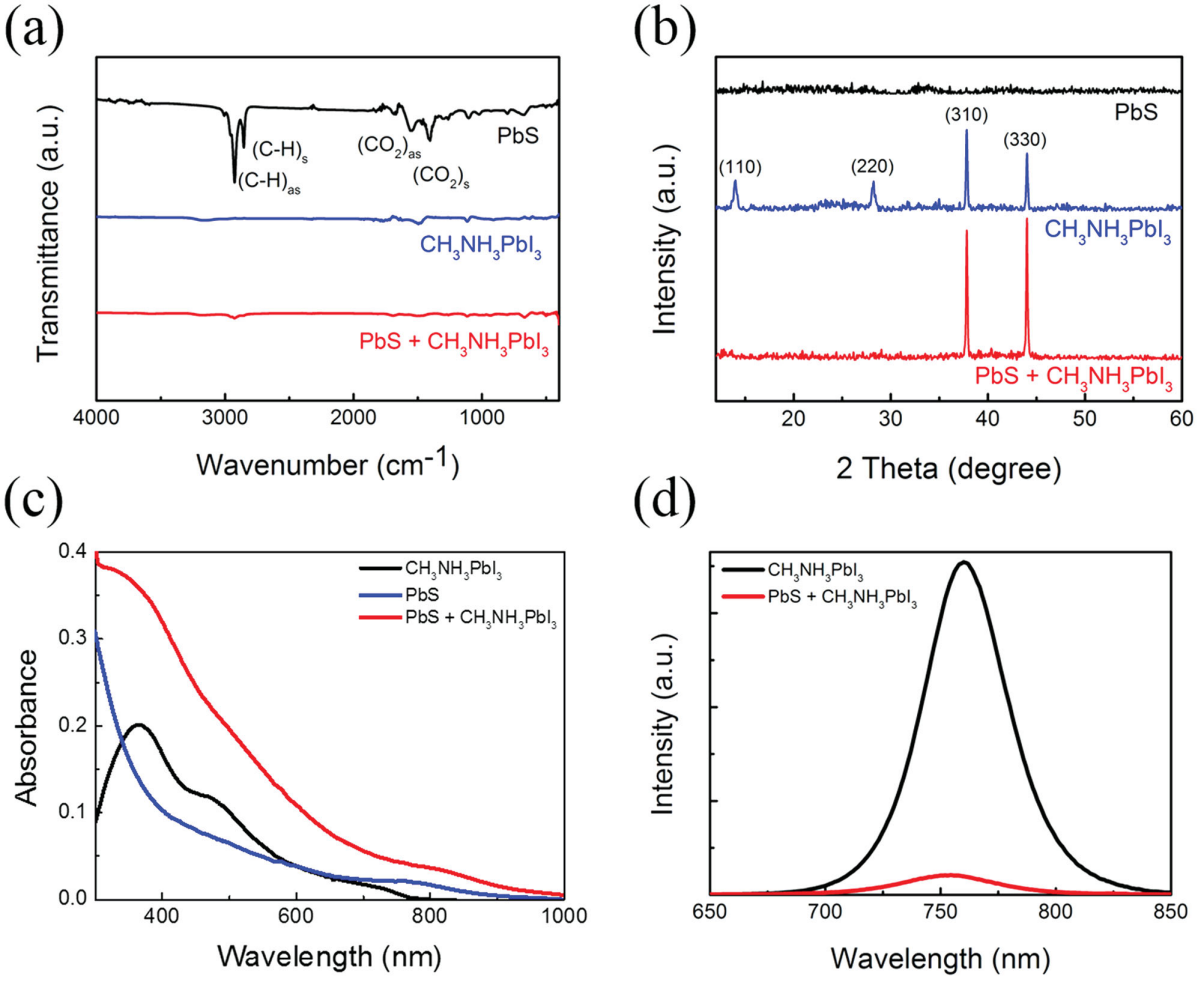
Characterization of ligand‐exchange process of PbS—CH_3_NH_3_PbI_3_ by comparing a) FTIR, b) XRD, c) optical absorption, and d) photoluminescence spectra of PbS, CH_3_NH_3_PbI_3_, and PbS—CH_3_NH_3_PbI_3_ CQDs.

X‐ray diffraction (XRD) measurement was then conducted to determine the crystalline structures of PbS—CH_3_NH_3_PbI_3_. As shown in Figure 2b, neat PbS CQDs have no obvious diffraction peaks due to low crystallization of 2.5 nm CQDs, while neat CH_3_NH_3_PbI_3_ film spin‐casted from acetonitrile solution shows strong peaks at 14.02°, 28.16°, 37.80°, and 44.02°, corresponding to the reflections from (110), (220), (310), and (330) lattice planes of typical CH_3_NH_3_PbI_3_ perovskite structure, respectively.^27^, ^28^ The PbS—CH_3_NH_3_PbI_3_ CQDs display strong characteristic peaks of CH_3_NH_3_PbI_3_ at 37.80° and 44.02°, further confirming the existence of CH_3_NH_3_PbI_3_. However, the intensity ratio of (310) and (330) peaks in PbS—CH_3_NH_3_PbI_3_ is lower than that of neat CH_3_NH_3_PbI_3_, implying different crystalline structures of perovskite caused by the interaction between CH_3_NH_3_PbI_3_ and PbS. Note that there is no diffraction peak assigned to PbI_2_ in both neat CH_3_NH_3_PbI_3_ and PbS—CH_3_NH_3_PbI_3_ CQDs, which confirms the presence of CH_3_NH_3_PbI_3_ ligand. The XRD results are further supported by the grazing‐incident wide‐angle X‐ray scattering (GIWAXS) measurement (Figure S4 in the Supporting Information), showing that all the characteristic peaks of both PbS and CH_3_NH_3_PbI_3_ retain in PbS—CH_3_NH_3_PbI_3_ CQDs with enhanced crystallinity.

X‐ray photoelectron spectroscopy (XPS) was subsequently applied to further ensure the presence of both PbS and CH_3_NH_3_PbI_3_ in the final film after the solid‐state ligand exchange (Figure S5 in the Supporting Information). Through the elemental analysis of XPS, it is estimated that the molar ratio of CH_3_NH_3_PbI_3_ ligand to PbS nanocrystals is about 1:3.7 after ligand exchange, in good agreement with that of solution‐phase ligand exchange of halometallate‐capped CQDs.^23^ The XPS results are also consistent with those of PbS‐in‐perovskite solids by in situ epitaxial growth process.^22^

Improved optical and electronic properties are anticipated in the PbS—CH_3_NH_3_PbI_3_ CQDs. As shown in Figure 2c, PbS CQDs exhibit a wide but low absorption range of 300−1000 nm, while CH_3_NH_3_PbI_3_ displays a strong but narrow absorption range from 300 to 760 nm. As expected, PbS—CH_3_NH_3_PbI_3_ indeed shows a broader and overall stronger absorption spectrum than neat films of CH_3_NH_3_PbI_3_ and PbS, respectively. Complementary optical absorption are thus achieved in such PbS—CH_3_NH_3_PbI_3_ in which the absorbance of CH_3_NH_3_PbI_3_ mainly contributes in the visible wavelength while PbS in the near‐infrared wavelength.

Photoluminescence (PL) and electrical conductivity measurements were then combined to study the charge transport between the CH_3_NH_3_PbI_3_ ligand and PbS. As shown in Figure 2d, a high PL quenching efficiency of ≈94% is found in the PbS−CH_3_NH_3_PbI_3_ compared to neat CH_3_NH_3_PbI_3_ film, indicative of effective charge transfer between PbS and CH_3_NH_3_PbI_3_. In addition, the emission peak of PbS—CH_3_NH_3_PbI_3_ blueshifts 6 nm than that of CH_3_NH_3_PbI_3_, suggesting the strong interaction between CH_3_NH_3_PbI_3_ and PbS, which is consistent with the XRD results. Moreover, electrical conductivity of neat PbS CQDs having mixed ligands of oleate and oleylamine is measured as only 5.33 × 10^−11^ S cm^−1^, while PbS—CH_3_NH_3_PbI_3_ CQDs show an improved conductivity of 1.88 × 10^−9^ S cm^−1^ by two orders of magnitude (Figure S6 in the Supporting Information). This is presumably attributable to enhanced charge transport of perovskite ligands and decreased inter‐particle spacing between PbS.

Lastly, PbS—CH_3_NH_3_PbI_3_ CQDs, which exhibit high conductivity and broad absorption ranging from UV to NIR wavelength, were investigated in CQDs solar cells as shown in **Figure** **3**. The device configuration of SnO_2_:F (FTO)/compact TiO_2_/several layers of PbS—CH_3_NH_3_PbI_3_/MoO_3_/Au and corresponding energy bandgap diagram are displayed in Figure 3a,b, respectively. Both the valence and conduction bands of CH_3_NH_3_PbI_3_ are lower than those of PbS, forming a suitable p–n heterojunction to facilitate charge separation. Additionally, it has proven that photoexcited electrons could transfer efficiently from quantum dots to TiO_2_ only for quantum‐dot diameter below 4.3 nm.^29^ In our work, the average diameter of PbS CQDs is 2.5 nm, indicating that charge transfer occurs between PbS and TiO_2_. CH_3_NH_3_PbI_3_ ligands can also act as an energy relay to form the cascade energy alignment between PbS and TiO_2_, which assists in reducing energy loss and facilitating charge transport.

**Figure 3:**
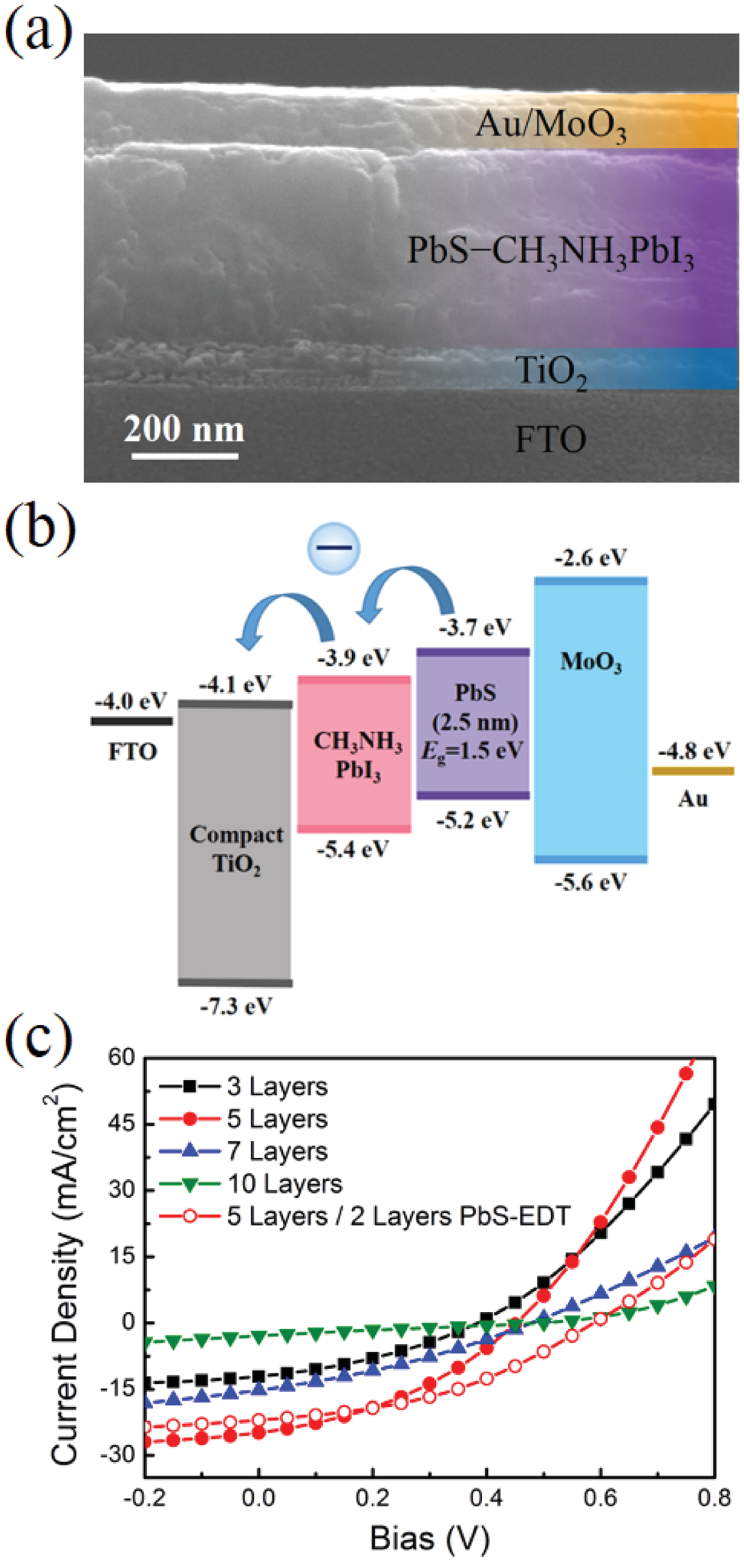
PbS—CH_3_NH_3_PbI_3_ CQDs‐based solar cells. a) Cross‐sectional SEM image of 5‐layer PbS—CH_3_NH_3_PbI_3_ CQD solar cells, and the corresponding b) energy bandgap diagram. Representative photocurrent density–voltage characteristics of c) single‐layer solar cells with different layer numbers of PbS—CH_3_NH_3_PbI_3_ CQDs, and bilayer solar cells with optimal 5‐layer PbS—CH_3_NH_3_PbI_3_ and 2‐layer PbS—EDT CQDs under AM 1.5G simulated light irradiation.

The number of the PbS—CH_3_NH_3_PbI_3_ layers on the TiO_2_ is anticipated to greatly influence the photovoltaic performance. Thin active layer shows poor light harvesting, while if the active layer is too thick, serious recombination would occur because of the limited carrier diffusion length of photogenerated charge carriers. The photocurrent density versus voltage characteristics (*J*−*V*) of the optimal PbS—CH_3_NH_3_PbI_3_‐based solar cells with different layer numbers under AM1.5G irradiation are shown in Figure 3c and summarized in **Table** **1**. The average values of photovoltaic parameters with a standard deviation of 45 individual devices are presented in Table S1 in the Supporting Information. Note that the PCE of solar cells fabricated from either single layer of neat CH_3_NH_3_PbI_3_ or pure PbS CQDs approaches zero because of the low concentration of CH_3_NH_3_PbI_3_ in acetonitrile and the existence of insulating ligands in PbS, respectively. As shown in Figure 3c and Table 1, among the devices with active layer numbers of 3, 5, 7, and 10, respectively, the five‐layer device exhibits an optimum PCE of 4.25% with a *J*_sc_ of 24.83 mA cm^−2^, an open‐circuit voltage (*V*_oc_) of 0.45 V, and a fill factor (FF) of 38%. Note that this PCE is significantly higher than those reported values of traditional EDT‐capped PbS solar cells (≈3%).^7^, ^8^, ^24^, ^25^ It is also notably higher than those of EDT capped PbS/CH_3_NH_3_PbI_3_ core/shell QDs sensitized solar cells (3.2%)^30^ and CH_3_NH_3_PbI_3_/oleic acid capped PbS bilayer heterojunction photovoltaic cells (3.6%),^31^ respectively.

**Table 1:** Summary of the optimal photovoltaic performance of PbS—CH_3_NH_3_PbI_3_ CQDs‐based solar cells with different layer numbers

Layers	PCE [%]	*J*_SC_ [mA cm^−2^]	*V*_OC_ [V]	FF [%]
3	1.60	12.11	0.40	33
5	4.25	24.83	0.45	38
5a) + 2b)	5.28	22.00	0.60	40
7	2.36	15.21	0.50	31
10	0.34	2.92	0.50	23

^a)^PbS—CH_3_NH_3_PbI_3_ CQDs;

^b)^PbS−EDT CQDs.

The thickness of 5‐layer PbS—CH_3_NH_3_PbI_3_ is measured as ≈350 nm from cross‐sectional SEM image in Figure 3a. It is thus plausible that deposition of five layers is a reasonable compromise to satisfy both the light absorption and carrier collection. The *V*_oc_ lies between 0.4 and 0.5 V, irrespective of the number of deposition layers, suggesting that the band structure of PbS—CH_3_NH_3_PbI_3_ solar cells is nearly identical to that of traditional PbS CQD solar cells.^7^ Moreover, the *J*_sc_ is among the highest values of PbS CQD solar cells,^5^, ^9^ which is presumably caused by complementary optical absorption, improved electrical conductivity, and efficient charge separation as a result of the forming p–n junctions between PbS and CH_3_NH_3_PbI_3_. Such combined benefits overcome the issues of insulating ligands of EDT and oleic acid in literature reports,^7^, ^8^, ^25^, ^26^, ^30^, ^31^ which largely hindered charge transport between PbS and CH_3_NH_3_PbI_3_. Also, owing to the relatively low concentration of CH_3_NH_3_PbI_3_ ligand in PbS—CH_3_NH_3_PbI_3_ CQDs as indicated by the XPS results, the resulting *J*_SC_ is primarily contributed by PbS CQDs and further enhanced by CH_3_NH_3_PbI_3_ ligands in visible light wavelength. The remarkably high *J*_sc_ is further supported by the external quantum efficiency (EQE) curve in Figure S7 in the Supporting Information. The maximum EQE of the optimal five‐layered device reaches 83% at 350 nm, and the wavelength covers broadly from 300 to 1000 nm. The photocurrent density calculated from the EQE spectrum is lower than *J*_SC_ possibly due to the trap states caused by the defects in TiO_2_ layer, which can be however filled by additional light soaking during *J*−*V* measurement. Finally, we anticipate that the relatively low FF can be further improved by interfacial modification, substitution of transporting layer, and application of pyramid‐patterned substrates/electrodes.^5^, ^9^

Recent studies showed an effective strategy of improving FF and PCE in the PbS CQD solar cells by inserting a thin top layer of PbS−EDT.^24^ We therefore added two layers of p‐type PbS−EDT CQDs (≈50 nm)^24^ onto the optimal five layers of n‐type PbS−CH_3_NH_3_PbI_3_ CQDs (≈300 nm) in solar cells to create graded band structures. As shown in Figure 3c and Table 1, the resulting bilayer devices reach a remarkably high PCE of 5.28% compared to single‐layer PbS—CH_3_NH_3_PbI_3_ cells. Such improvement is mainly reflected in enhanced *V*_OC_ (0.6 V) and increased FF (40%), although slightly lowering the *J*_SC_. To interpret it, we attempted to investigate the influence of the PbS−EDT layer on transport dynamics by time‐of‐flight (ToF) measurements, as shown in Figure S8 in the Supporting Information. Without PbS−EDT layer, the hole and electron mobilities are 8.04 × 10^−5^ and 2.68 × 10^−5^ cm^2^ Vs^−1^, respectively. The low and imbalanced charge transport explains the low FF obtained in the single‐layer device. After the addition of PbS−EDT layer, the electron mobility is increased by an order of magnitude to 1.04 × 10^−4^ cm^2^ Vs^−1^ while the hole mobility is slightly increased to 9.91 × 10^−5^ cm^2^ Vs^−1^. It can be thus seen that the PbS−EDT layer enables more balanced and efficient charge transport in the device.

In conclusion, we have successfully fabricated CH_3_NH_3_PbI_3_ perovskite capped PbS CQDs by solid‐state ligand exchange method. By using acetonitrile solvent in this process, original mixed ligands of oleate and oleylamine have been fully replaced by CH_3_NH_3_PbI_3_. Compared to original PbS CQDs, the PbS—CH_3_NH_3_PbI_3_ CQDs exhibited complementary optical absorption spectra, facile charge separation, increased conductivities, and efficient charge transport. Through layer‐by‐layer process, PbS—CH_3_NH_3_PbI_3_ CQDs‐based inverted solar cells with various deposition layers were made. The best solar cell gave a high PCE of 4.25% with an impressive *J*_sc_ of 24.83 mA cm^−2^. Furthermore, by inserting a top layer of EDT‐capped PbS CQDs, the resulting bilayer solar cells reached a significantly enhanced PCE of 5.28%, which is ascribed to more balanced and efficient charge transport in the device.

## Supporting information

As a service to our authors and readers, this journal provides supporting information supplied by the authors. Such materials are peer reviewed and may be re‐organized for online delivery, but are not copy‐edited or typeset. Technical support issues arising from supporting information (other than missing files) should be addressed to the authors.

Supplementary
