# Supplementary material for: Solid‐State Ligand‐Exchange Fabrication of CH3NH3PbI3 Capped PbS Quantum Dot Solar Cells
Source: Adv Sci (Weinh). 2016 Feb 18;3(6):1500432. doi: 10.1002/advs.201500432 (PMC5067684; doi:10.1002/advs.201500432)
Supplement: Supplementary file 1 — Supplementary [file ADVS-3-0d-s001.pdf]

Copyright WILEY-VCH Verlag GmbH & Co. KGaA, 69469 Weinheim, Germany, 2016.

ADVANCED SCIENCE
Open Access

Supporting Information

for *Adv. Sci.*, DOI: 10.1002/advs. 201500432

Solid-State Ligand-Exchange Fabrication of $CH_3NH_3PbI_3$ Capped PbS Quantum Dot Solar Cells

*Jiajun Peng, Yani Chen, Xianfeng Zhang, Angang Dong, and Ziqi Liang**

Solid-State Ligand-Exchange Fabrication of $CH_3NH_3PbI_3$ Capped PbS Quantum Dot Solar Cells

*Jiajun Peng,[1] Yani Chen,[1] Xianfeng Zhang,[2] Angang Dong,[2] and Ziqi Liang[1]**

[*] [1]Prof. Z. Liang, J. Peng, Y. Chen
Department of Materials Science, Fudan University
Shanghai 200433, China
Email: zqliang@fudan.edu.cn

[2]Prof. A. Dong, X. Zhang
Department of Chemistry, Fudan University
Shanghai 200433, China

Experimental Section

Materials: All chemical reagents were purchased from J&K Scientific, Ltd. (China) and used without further purification. All the solvents used for the synthesis were HPLC grade. Gold (99.99%) was purchased from Zhong Nuo Advanced Materials (Beijing) Technology Co., Ltd. and used as received.

Synthesis: PbS nanocrystals were synthesized according to the method of Hines *et al.* with slight modifications.[1] For obtaining PbS CQDs with a bandgap 1.5 eV, a mixture of oleic acid (1.34 g, 4.8 mmol), oleylamine (0.134 g,1.5 mmol), PbO (0.45 g, 2.0 mmol), and 1-octadecene (ODE) (14.2 g, 56.2 mmol) was heated to 150 °C under vacuum for one hour before placing under N_2. The flask temperature was decreased to 110 °C and the TMS/ODE mixture (0.18 g, 1 mmol in 10 mL) was injected rapidly. After injection, the temperature dropped to ~85 °C, and the flask was allowed to cool gradually to room temperature. The nanocrystals were precipitated with 100 mL of acetone/ethonal (1:1) mixture and centrifuged. After discarding the supernatant, the precipitate was redispersed in hexane. The centrifugation process was repeated twice, and finally the nanocrystals were redispersed in octane at a concentration of 20 mg/mL.

Characterization: Optical absorption and photoluminescence spectra of samples were acquired on Shimadzu UV 3600 spectrophotometer/Agilent 8453 UV-Visible spectrophotometer and a Horiba FluoroMax®-4 spectrofluorometer, respectively. Fourier-transform infrared spectroscopy was measured by PerkinElmer Spectrum Two. X-ray diffraction pattern data for 2θ values were collected with a Bruker AX D8 Advance diffractometer with nickel filtered Cu Kα radiation (λ = 1.5406 Å). Grazing incident wide-angle X-ray scattering (GIWAXS) measurements were performed at the beamline BL14B1 with energy of 10 kV, a wavelength of 1.2398 nm and a pixel size of 73.2419 μm in Shanghai

Synchrotron Radiation Facility (SSRF). X-ray photoelectron spectroscopy experiments were carried out on a RBD upgraded PHI-5000C ESCA system (Perkin Elmer) with Mg K_{α} radiation (hν=1253.6 eV). Binding energies were calibrated by using the containment carbon (C_{1s} = 284.6 eV). Transmission electron microscopy imaging and energy dispersive X-ray (EDX) analysis were conducted on a FEI Tecnai G2 F20 S-TWIN electron microscope operating at an accelerating voltage of 200 kV. Cyclic voltammetry measurement was done with Model 660E Series Potentiostat/Galvanostat (CH Instruments, Inc.) interfaced to a PC. For the electrical conductivity measurement, pure PbS and $CH_3NH_3PbI_3$ capped PbS CQDs were deposited on Pt interdigitated electrodes (IDEs) with an electrode spacing of 10 μm. Current−voltage (I−V) characteristics were acquired by sweeping between −1 and +1 V with a step size of 0.01 V in the dark. Scanning Electron Microscopy (SEM) images were acquired on a JEOL JSM-6701F field-emission SEM at an accelerating voltage of up to 30 kV.

Device Fabrication and Measurements: Patterned SnO_2:F (FTO) substrates (12 Ω/□, Thin Film Devices, Inc.) were cleaned sequentially in an ultrasonic solvent bath of deionized water, acetone, and isopropyl alcohol. Substrates underwent $TiCl_4$ treatment in a 0.2 M solution in deionized (DI) water at 70 °C for 30 min. Substrates were removed, rinsed with DI water, and heated at 520 °C for 40 min. The fabrication of CQD films was performed using a layer-by-layer (LbL) spin-coating technique on the FTO substrate. Solid-state ligand exchange was performed in N_2 atmosphere glovebox using saturated $CH_3NH_3PbI_3$ solution in acetonitrile. The fabrication process was as follows: (1) 100 μL of PbS CQDs in octane (20 mg/ml) were dropped onto the substrate through a 0.22 μm filter and were spin-cast at 2500 rmp for 20 s; (2) 300 μL of saturated $CH_3NH_3PbI_3$ solution in acetonitrile was drop-casted onto the entire PbS layer for 1 min through a 0.22 μm filter, followed by spin-coating at 2500 rmp for 20 s to remove the

excess solution; (3) two rinse steps were applied with acetonitrile and then octane, each followed by spin-drying at 2500 rmp for 20 s; (4) finally, the substrate was heated at 50 °C for 10 s. Step 1−4 were repeated several times until the desired film thickness was reached (3−10 layers). In the fabrication of bilayer solar cells, the depostion steps of additional EDT-capped PbS CQDs on PbS−$CH_3NH_3PbI_3$ CQDs are the same as the above except for Step 2, which is described as follows. A 300 μL of ethaneditiol (0.01 v/v %) in acetonitrile was drop-casted onto the entire PbS CQDs layer for 30 s, followed by spin-coating at 2500 rmp for 10 s to remove the excess solution.

The above samples were then loaded into a glovebox-integrated deposition chamber and pumped down to a pressure of $<10^{-4}$ Pa. A sequence of MoO_3 (10 nm) and Au (100 nm) layers were sequentially deposited by thermal evaporation through a shadow mask at a rate of 0.1 Å/s and 1 Å/s, respectively. The active area as defined shadow mask is ~0.04 cm^2. The sample was mounted inside a nitrogen-filled sample holder with a quartz optical window for subsequent measurements. The *J*–*V* data was acquired with a Keithley 2400 source–meter unit. The light *J*–*V* curves were measured under illumination with a Newport-Oriel (Sol3A Class AAA Solar Simulator, 94043A) AM 1.5G light source operating at an intensity of 100 mW·cm^{-2}. The light intensity was calibrated by a certified Oriel reference cell (91150V) and verified with a NREL calibrated, filtered silicon diode (Hamamatsu, S1787-04). External quantum efficiency (EQE) spectra were measured on a commercial EQE set-up (QE-R, Enli Technology Co., Ltd). A calibrated silicon diode with a known spectral response was used as a reference.

For TOF measurements, the sample was first mounted inside a nitrogen-filled sample holder with a quartz optical window, and then illuminated from the FTO side using the second harmonic (532 nm) of a Nd:YAG laser oscillator (Continuum Minilite II) with a 3−7 ns pulse

width to excite the organic layer and generate transient currents. The laser light intensity is 0.1 mJ/cm^2. The photogenerated charges drifted across the sample under the external electric field applied by a source-measure unit (Keithley 2400). The extracted photocurrents across the 50 Ω input impedance were recorded by the Tektronix Oscilloscope. Transient currents were acquired by averaging 64 frames. Voltage bias of 2 V are applied to measure the charge mobility of either hole or electron. The positive probe is connected to the Au electrode and the negative probe is connected to the FTO electrode. Positive bias are applied to measure hole mobility and negative bias are applied to measure electron mobility. The TOF results can be calculated from the following equation: $\mu = \frac{d^2}{V t_{tr}}$, where μ is the charge mobility, d is the thickness of the active layer, V is the voltage bias, and t_{tr} is the transient time.

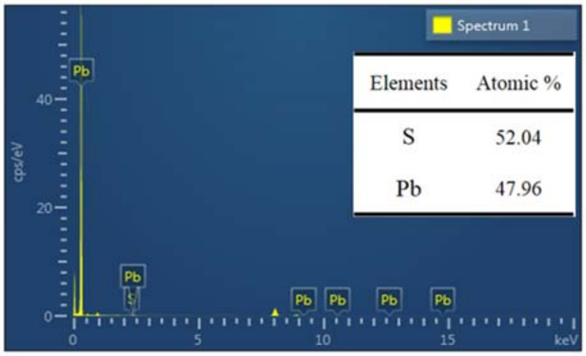

Figure S1. Energy dispersive X-ray (EDX) analysis of PbS CQDs.

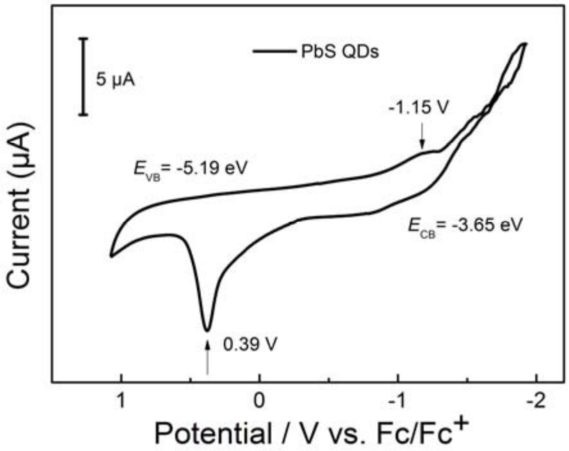

Figure S2. Cyclic voltammetry data of PbS CQDs on Pt electrode in an acetonitrile solution of 0.1 mol/L Bu_4NPF_6 at a scan rate of 50 mV/s.

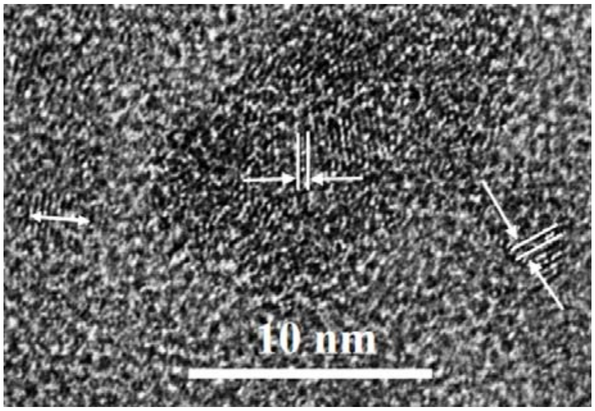

Figure S3. TEM characterization of $CH_3NH_3PbI_3$ capped PbS CQDs.

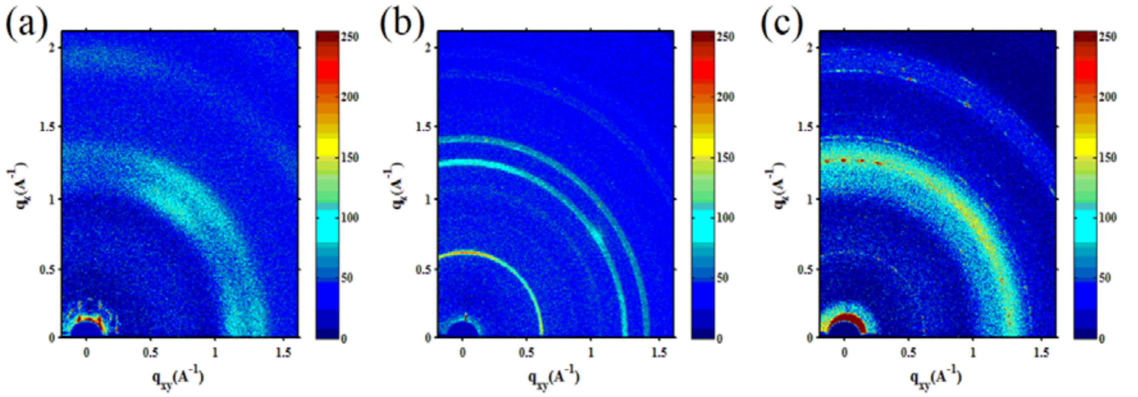

Figure S4. GIWAXS patterns of (a) PbS CQDs, (b) $CH_3NH_3PbI_3$ and (c) PbS−$CH_3NH_3PbI_3$ CQDs.

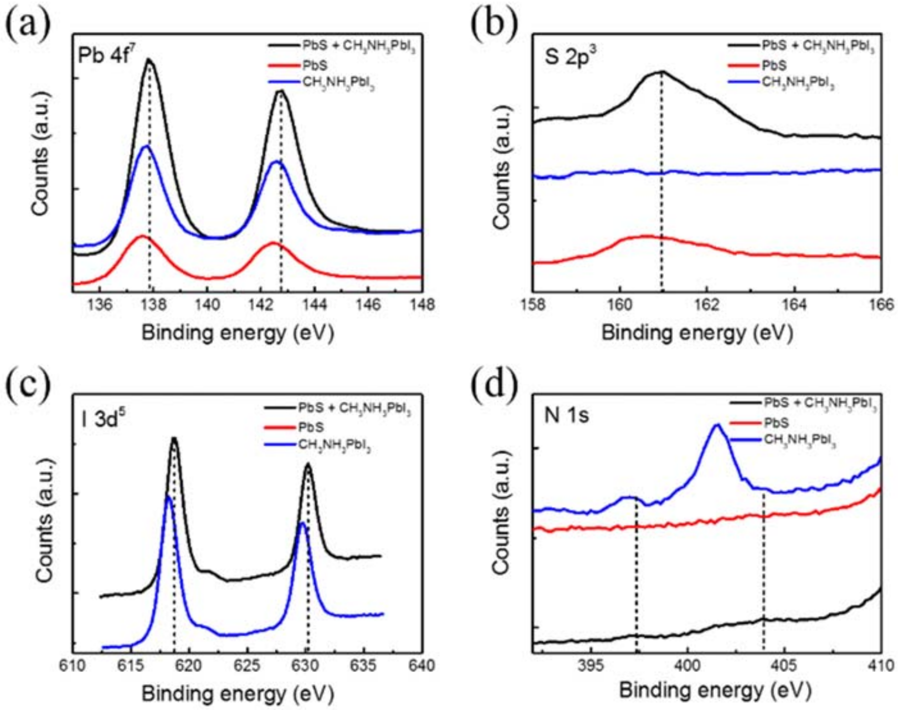

Figure S5. XPS spectra of (a) Pb 4f[7], (b) S 2p[3] (c) I 3d[5] and (d) N 1s peaks in PbS CQDs, $CH_3NH_3PbI_3$ and PbS−$CH_3NH_3PbI_3$ CQDs, respectively.

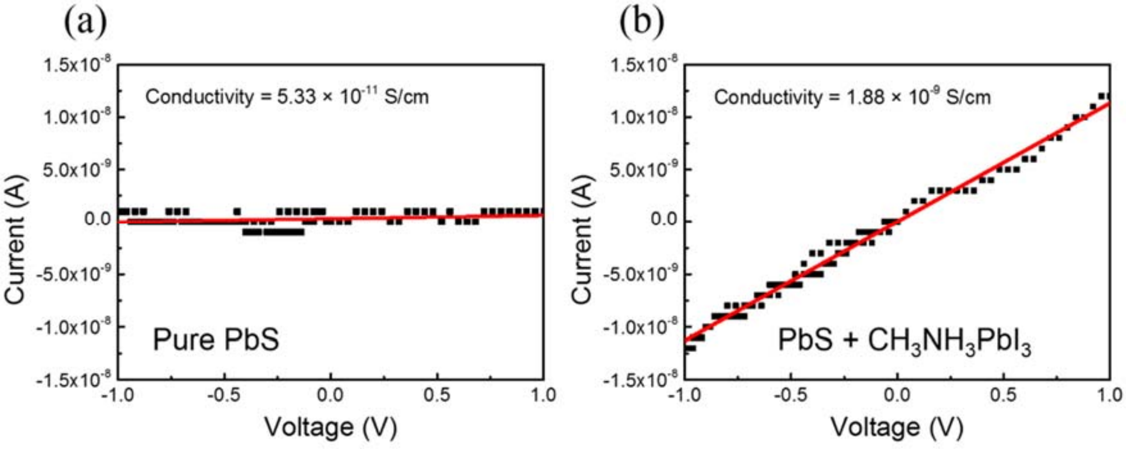

Figure S6. Current-voltage curves of (a) pure PbS CQDs and (b) PbS−$CH_3NH_3PbI_3$ CQDs.

Table S1. Summary of average photovoltaic performance of 45 individual PbS−$CH_3NH_3PbI_3$ CQDs based solar cells with different layer numbers

Layers	PCE (%)	J_{SC} (mA/cm^2)	V_{OC} (V)	FF (%)
3	1.32 ± 0.28	10.03 ± 2.08	0.40 ± 0.01	33 ± 1
5	4.15 ± 0.10	25.20 ± 0.71	0.45 ± 0.01	37 ± 1
$5^a + 2^b$	5.19 ± 0.09	22.06 ± 0.17	0.60 ± 0.01	39 ± 1
7	2.30 ± 0.06	14.62 ± 0.59	0.48 ± 0.02	34 ± 4
10	0.30 ± 0.04	2.72 ± 0.28	0.48 ± 0.02	22 ± 1

Note: ^{a} PbS−$CH_3NH_3PbI_3$ CQDs, ^{b} PbS−EDT CQDs

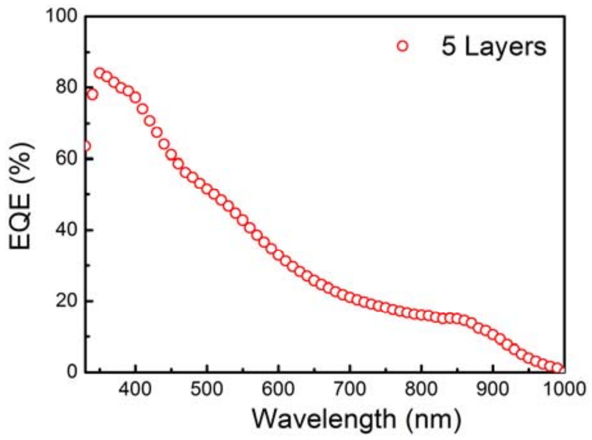

Figure S7. EQE spectrum of the optimal 5-layer PbS−$CH_3NH_3PbI_3$ solar cells.

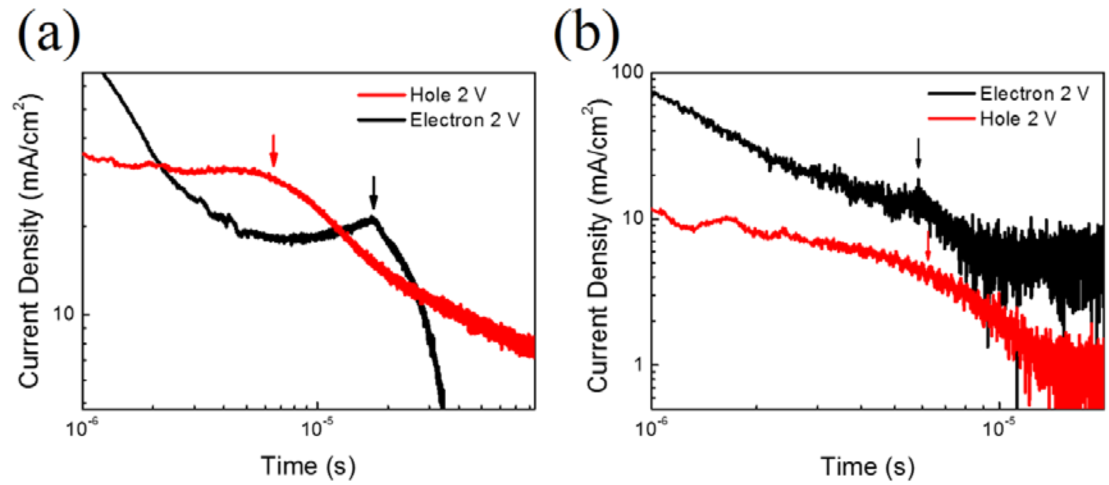

Figure S8. Comparison of the current density versus time profiles by TOF measurements between (a) PbS−$CH_3NH_3PbI_3$ CQDs based single-layer solar cells and (b) PbS−$CH_3NH_3PbI_3$ / PbS−EDT CQDs based bilayer solar cells. Each t_{tr} is indicated by the arrow.
